# Temporal changes in circulating metabolites after metabolic and bariatric surgery and risk of incident coronary heart disease: evidence from prospective cohort and nested case–control studies

**DOI:** 10.1097/JS9.0000000000005085

**Published:** 2026-06-03

**Authors:** Yulu Zheng, Zicheng Wang, Lei Wang, Charles R. Flynn, Xiao-Ou Shu, Wayne J. English, Jason M. Samuels, You Chen, Loren Lipworth, Deepak Gupta, Qiuyin Cai, Wei Zheng, Danxia Yu

**Affiliations:** a Division of Epidemiology, Department of Medicine, Vanderbilt University Medical Center, Nashville, Tennessee, USA; b Department of Surgery, Vanderbilt University Medical Center, Nashville, Tennessee, USA; c Department of Biomedical Informatics, Vanderbilt University Medical Center, Nashville, Tennessee, USA; d Division of Cardiovascular Medicine, Department of Medicine, Vanderbilt University Medical Center, Nashville, Tennessee, USA

**Keywords:** metabolomics, metabolic and bariatric surgery, incident coronary heart disease

## Abstract

**Background:**

Metabolic and bariatric surgery (MBS) has notable cardiovascular benefits beyond weight loss.

**Methods:**

The Gut Microbiome in Metabolic Surgery Study (GUMMY) enrolled patients who underwent first-time Roux-en-Y gastric bypass or sleeve gastrectomy, with blood samples collected at pre- and 3- and 12-month post-surgery. Significant metabolite changes were identified using paired *Wilcoxon* signed-rank tests with a false discovery rate (FDR) < 0.05 and |log_2_ fold change| > log_2_1.5, comparing 3-month post- vs. pre-surgery (early phase) and 12- vs. 3-month post-surgery (late phase). Significantly changed metabolites were classified into three patterns: early changed, late sustained; early changed, late reversed; and early unchanged, late changed. Subsequently, a nested case–control study within the Southern Community Cohort Study (SCCS) assessed the associations between surgery-altered metabolites and incident coronary heart disease (CHD) risk using conditional logistic regression. The same untargeted metabolomic assay was conducted in GUMMY and SCCS plasma samples.

**Results:**

Among 115 surgical patients, the mean (SD) age was 44.9 (9.5) years, and 90 (78%) were women. Significant changes were observed in 224 metabolites, mainly encompassing sustained increases in bile acids and decreases in branched-chain amino acids, lactoyl, and lysine amino acids; reversed changes in some ketone bodies, phospholipids, lysophospholipids, and glycogen metabolites; late increases in taurine, caffeine, benzoate, and some tryptophan metabolites; and late decreases in ceramide metabolites and certain fatty acids. In the SCCS (*n* = 1194; 597 case–control pairs), 37 surgery-altered metabolites were significantly associated with incident CHD (FDR < 0.1), 28 with concordant effects, i.e., post-surgical increases were associated with reduced CHD risk and vice versa.

**Conclusions:**

MBS elicits substantial metabolomic alterations, with many altered metabolites associated with incident CHD. These findings provide mechanistic insights into the cardiovascular benefits of MBS and suggest potential therapeutic targets for CHD prevention.

## Graphical abstract

http://links.lww.com/JS9/H489

## Introduction

Obesity, typically defined as a body mass index (BMI) of 30 kg/m^2^ or higher, is a critical global public health challenge. In the United States (U.S.), over 40% of adults are obese; meanwhile, the prevalence of severe obesity (BMI ≥40 kg/m^2^) doubled from 4.7% in 1999 to 9.7% in 2023^[^1–3^]^. Obesity, especially severe obesity, increases the risk for major chronic diseases, including cardiovascular disease (CVD), type 2 diabetes (T2D), certain cancers, and mental disorders, and is linked to higher cardiovascular mortality^[^4–6^]^. However, the causes and biological mechanisms underlying the development and treatment of obesity remain incompletely understood^[^5^]^.

Metabolic and bariatric surgery (MBS) is currently the most effective and durable treatment for severe obesity^[^6–8^]^. The most common procedures are Roux-en-Y gastric bypass (RYGB) and sleeve gastrectomy (SG), which achieve approximately 30% weight loss at 1 year and in the long term after surgery^[^7,9^]^. Beyond weight loss, patients experienced remarkable cardiometabolic improvements following these procedures, including reductions in blood pressure, non-high-density lipoprotein cholesterol, glucose, and hemoglobin A1c^[^6,10^]^. Moreover, MBS has been linked to a reduced incidence of coronary heart disease (CHD), heart failure, and stroke^[^11^]^. The cardiovascular benefits of MBS may be attributed to multiple pathways, including altered central appetite regulation, enhanced secretion of gut peptides (e.g., glucagon-like peptide-1, peptide YY), and changes in gut microbiota and bile acid circulation, which influence energy, glucose, and lipid metabolism^[^12–15^]^. The interplay between metabolomic profiling and metabolic regulation underscores the complex, multifactorial mechanisms underlying obesity management.

Comprehensive metabolomic profiling before and longitudinally after MBS may provide detailed insights into the mechanisms underlying the cardiovascular benefits and establish mechanistic links between surgical interventions and systemic physiological adaptations. Previous studies have demonstrated substantial post-MBS changes in metabolites, primarily involving lipids and amino acids^[^16,17^]^, such as bile acids^[^8,13,18,19^]^, fatty acids^[^20–23^]^, ceramides^[^24,25^]^, branched-chain amino acids (BCAAs; leucine, isoleucine, and valine)^[^26–29^]^, tryptophan metabolites^[^14^]^, and sulfur-containing metabolites^[^30^]^. However, critical research gaps remain. Previous studies were limited by small sample sizes (usually *n* < 50), relatively short follow-up periods (1-, 3-, or 6-month post-surgery), lack of analysis of temporal patterns, and/or lack of adjustment for potential confounders such as diet, medications, or baseline comorbidities. More importantly, to our knowledge, no studies have further examined whether and which MBS-altered metabolites are related to incident CHD, leaving the mechanistic link between MBS-induced metabolite changes and their potential long-term cardiovascular benefits unclear.

To address these gaps, we conducted a comprehensive two-phase study with several methodological advantages. First, we characterized temporal changes in over 1100 circulating metabolites among MBS patients (*n* = 115) at baseline (pre-surgery), 3-month post-MBS, and 12-month post-MBS. We applied stringent criteria for statistical significance and effect size to identify temporal changes from baseline to 3 months and from 3 to 12 months, thereby distinguishing transient surgical responses from potential sustained metabolic adaptations. Second, we extended our investigation by examining associations between surgery-altered metabolites and incident CHD risk in an independent nested case–control study (*n* = 1,194) using the same metabolomic platform. This integrated approach allowed us to elucidate both the temporal dynamics of metabolite changes after MBS and their potential implications for long-term cardiovascular outcomes. This research, involving a prospective cohort study and an independent nested case–control study, has been reported in line with the STROCSS guidelines^[^31^]^.

## Methods

### Study population and design

The Gut Microbiome in Metabolic Surgery Study (GUMMY) is an ongoing cohort of patients who underwent first-time RYGB or SG at a medical center in the Southeastern U.S., aiming to investigate longitudinal multi-omics changes after MBS. Patients were included if they: (1) aged 20–65 years; (2) having at least one obesity-related metabolic condition, i.e., prediabetes or T2D, hypertension, and dyslipidemia; and (3) were willing to provide written informed consent, survey-based information, and biospecimens. Exclusion criteria include prior gastric operations; prior CVD (CHD, stroke, or heart failure); prevalent gastrointestinal disease (e.g., inflammatory bowel disease or celiac disease); chemo/radiation therapy for cancer within 2 years; and vomiting, constipation, or diarrhea within 7 days before enrollment. The present study included the first 115 GUMMY participants enrolled between September 2021 and August 2023.

HIGHLIGHTS

- MBS-induced metabolite changes follow distinct temporal patterns.
- MBS-altered metabolites are significantly associated with incident CHD risk.
- Specific amino acids, lipids, and xenobiotics show concordant effects between post-surgical changes and incident CHD risk.
- Findings reveal potential metabolomic mechanisms underlying the cardiovascular benefits of MBS.

Significantly altered metabolites identified in GUMMY were further investigated for their associations with incident CHD in a case–control study nested within the prospective Southern Community Cohort Study (SCCS). Details of the design and protocol for the SCCS have been described elsewhere^[^32^]^. Briefly, the SCCS recruited 84 735 study participants (aged 40–79 years) from 12 southeastern states in the U.S. in 2002–2009. The SCCS collected baseline biospecimens and followed participants for disease outcomes and vital status. Participants were eligible for the nested case–control study if they (1) had no baseline history of CVD, cancer, or end-stage renal disease; (2) provided a plasma sample with documented fasting and processing times; (3) reported no antibiotic or cold/flu medication use within 7 days before blood collection; and (4) had Centers for Medicare & Medicaid Services (CMS) coverage with ≥2 claims after SCCS enrollment through December 2016. CHD cases were identified through CMS claims using validated ICD-9/-10 codes, as published in our previous article, and through the National Death Index, with CHD listed as the underlying cause of death^[^33^]^. Cases were 1:1 matched with controls on race, sex, enrollment age (±2 years), fasting time (±2 hours), and sample processing time (±4 hours). Both GUMMY and SCCS were approved by the respective institutional review boards (IRBs), with IRB approval numbers provided in the *Author Disclosure Form*. The nested case–control study within the SCCS was approved by the SCCS Data and Biospecimen Use Committee. An overview of the study design is presented in Supplemental Digital Content Figure 1, available at: http://links.lww.com/JS9/H486.

### Metabolites profiling

Plasma samples were shipped on dry ice to Metabolon Inc. (Morrisville, NC, USA). Untargeted metabolomics profiling was performed using ultra-high-performance liquid chromatography–tandem mass spectrometry, following published standard protocols^[^34,35^]^. All pre- and post-surgery samples from each patient were placed adjacent to each other in random order within the same batch, as were baseline plasma samples from matched CHD case–control pairs; laboratory personnel were blinded to the pre-/post-surgery or CHD case–control status of the samples. This design inherently minimizes batch effects in our analyses comparing within-person pre-to-post-surgery metabolite changes and metabolite differences in matched case–control pairs. Plasma samples were extracted with methanol and split into four aliquots for analysis in both positive and negative ion modes using a combination of reverse-phase and hydrophilic interaction chromatography methods. Quality control was ensured through internal standards and technical replicates, with a median relative standard deviation of 5% for instrument variability and 8% for overall process variability. Metabolites were identified by automated comparison of mass spectra features to a reference library of >4000 authenticated standard compounds, followed by visual inspection for quality control. Most metabolites (>80%) were annotated based on internal standards. Metabolites annotated only by a match to a known mass spectrum or chemical formula were marked by “*” and “**,” respectively. Peaks were quantified using the area-under-the-curve. A total of 1442 metabolites were detected in GUMMY; 1503 metabolites were detected in the nested case–control study. After excluding metabolites with >50% missing values across all three time points in GUMMY and removing the metabolites with >90% missing values across participants in the CHD case–control study, a total of 1199 and 1322 metabolites were kept, respectively. Missing value proportions for 1442 metabolites in GUMMY at each time point are provided in Supplemental Digital Content Table 1, available at: http://links.lww.com/JS9/H488. Metabolites processing and quality control procedures in SCCS have been described previously^[^33^]^. Metabolites with missing values were imputed with half the minimum value in the non-missing samples, a standard approach in metabolomics research, as missing values in metabolomics data predominantly reflect very low levels below the limit of detection^[^36,37^]^. The values of all metabolites were log-transformed and standardized to mean 0 and unit variance before statistical analysis.

### Statistical analysis

Participants’ characteristics were summarized as mean (SD) for continuous variables and frequency (%) for categorical variables. The changes in circulating metabolites after MBS were determined by paired Wilcoxon signed-rank tests along with fold change (FC). To ensure both statistical significance and at least moderate-to-large effect sizes, the Benjamini–Hochberg false discovery rate (FDR) with *P* < 0.05 and |log_2_ FC| > log_2_ 1.5 (i.e., FC > 1.5 or < 0.67) were applied to categorize three patterns of significant changes: (1) early changed, late sustained (i.e., significant changes when comparing 3-month post- to pre-surgery, which did not change significantly when comparing 12- to 3-month post-surgery); (2) early changed, late reversed (significant changes in opposite directions from pre- to 3-month and from 3- to 12-month post-surgery); and (3) early unchanged, late changed (only significant when comparing 12- to 3-month post-surgery). Metabolites with significant changes in GUMMY were then evaluated for their associations with incident CHD in the nested case–control study within the SCCS, using conditional logistic regression. Model 1 was adjusted for age, while Model 2 was the primary model with additional adjustments for educational attainment, income, smoking status, alcohol intake, physical activity, and diet quality (see the legend of Supplemental Digital Content Table 1, available at: http://links.lww.com/JS9/H488).

A series of sensitivity and alternative analyses was conducted. First, linear mixed models (LMMs) were performed in GUMMY, with metabolite levels as the dependent variable and continuous time trajectory (per 3 months) as the independent variable. Two LMMs were established: one without adjustment and one adjusted for demographic factors (age, sex, race), procedure type, baseline comorbidities (diabetes, hypertension, dyslipidemia), having antibiotics or bowel preparations within 2 months, diet quality, fasting time, and baseline weight. Model coefficients were compared to evaluate the confounding effects of those covariates. Second, to examine potential effect modification, we added an interaction term between time and each of the following variables: sex, race, and procedure type to the fully adjusted LMM. Third, we identified additional moderately changed metabolites after MBS using relaxed FC thresholds (FDR < 0.05; |log₂FC| > log₂1.2). Fourth, when evaluating associations between metabolites and incident CHD in SCCS, we further adjusted for BMI, history of diabetes, history of hypertension, and history of dyslipidemia (Model 3). Fifth, we evaluated potential effect modification by race, sex, diabetes, hypertension, dyslipidemia, and obesity on the metabolite–CHD associations in SCCS, adjusting for the interaction effect of corresponding factors and metabolite levels, as per Model 2. For metabolites with significant interaction effects, we conducted stratified analyses using conditional or unconditional logistic regression with adjustment for potential confounding factors in Model 2 and matching factors when applicable (Supplementary Legends). All analyses were performed using R (version 4.4.1).

## Results

### Characteristics of study participants

GUMMY participants had a mean age of 44.9 ± 9.5 years, were predominantly female (78%), and White (85%), with a mean pre-surgical BMI of 46.2 ± 6.8 kg/m^2^ and a high prevalence of diabetes, dyslipidemia, and hypertension (57–84%). At 12-month post-surgery, patients exhibited substantial reductions in body weight (−30.6%), glucose (−16.7 mg/dL), and blood pressure (systolic: −11.1 mmHg; diastolic: −4.8 mmHg) (Table 1). In SCCS, 597 case–control pairs were included; incident CHD cases had a mean (SD) age of 55.0 (8.7) years at baseline and a median follow-up of 5 years (interquartile range: 3–8). Incident CHD cases were characterized by higher BMI (30.96 ± 7.27 vs. 29.67 ± 7.18 kg/m^2^ in controls) and greater prevalence of diabetes, hypertension, and dyslipidemia compared to their age-, sex-, and race-matched controls (Supplemental Digital Content Table 2, available at: http://links.lww.com/JS9/H488).

**Table 1:** Characteristics of GUMMY participants (*n* = 115).

Characteristics *	Baseline (pre-surgery)	3 months after surgery	12 months after surgery	*P*-value
Number of patients (counts)	115	101	112	
Age [years, mean (SD)]	44.9 (9.5)			
Sex (female, %)	90 (78.3)			
Self-identified race				
White	98 (85.2)			
Black	17 (14.8)			
Procedure type				
RYGB	59 (51.3)			
VSG	56 (48.7)			
Educational attainment				
Less than college	46 (40.4)			
College or higher	68 (59.6)			
Ever smoked ≥ 100 cigarettes	42 (36.8)			
Regular alcohol drinking^a^	18 (15.7)			
History of diabetes^b^	69 (60.0)			
History of dyslipidemia^b^	65 (56.5)			
History of hypertension^b^	97 (84.3)			
Diet quality^c^				
Low	70 (60.9)	63 (62.4)	67 (59.8)	0.300
Middle	37 (32.2)	24 (23.8)	29 (25.9)	
High	8 (7.0)	14 (13.9)	16 (14.3)	
Weight [kg, mean (SD)]	130.7 (22.9)	109.3 (20.4)	90.8 (19.0)	**<0.001**
BMI [kg/m^2^, mean (SD)]	46.15 (6.76)	39.14 (6.52)	32.62 (6.07)	**<0.001**
Blood glucose [mg/dL, mean (SD)]	108.80 (37.57)	100.40 (38.19)	92.06 (22.41)	**0.004**
Systolic blood pressure [mmHg, mean (SD)]	132.64 (12.57)	124.54 (13.34)	121.52 (10.73)	**<0.001**
Diastolic blood pressure [mmHg, mean (SD)]	77.93 (9.70)	73.05 (11.29)	73.13 (9.71)	**<0.001**
Fasting hours before blood work [mean (SD)]	8.89 (5.93)	9.04 (6.19)	8.16 (5.80)	0.544
Antibiotics or bowel preparations within 2 months	16 (13.9)	10 (9.9)	11 (9.8)	0.542

SD, standard deviation; RYGB, Roux-en-Y gastric bypass; VSG, sleeve gastrectomy.

^*^ Characteristics are presented as *n* (%) unless otherwise specified.

Bold values represent statistically significant results (*P* < 0.05).

Clinical and biochemical characteristics of individuals in each timepoint are included. Comparison across pre-surgery, 3-, and 12-month post-surgery were made using repeated-measures ANOVA (for weight, BMI, SBP, DBP, blood glucose, fasting hours before blood work) and Chi-squared tests (for diet quality).

^a^ Regular alcohol was defined as self-reported alcohol (beer, wine, liquor) intake >2 times a week.

^b^ Histories of diabetes, dyslipidemia, and hypertension were determined by self-reported doctor diagnosis or medication use.

^c^ Diet quality was evaluated by a 7-point binary scale was defined based on “Your Med Diet Score.” Points were awarded (1 for “yes”) for meeting the criteria: daily consumption of ≥2 servings of vegetables, fruits, or whole grains; weekly consumption of ≥2–3 servings of ocean fish, beans/lentils, or nuts; and <2 servings per week of processed or red meat. Diet quality was categorized as low (≤2), medium (3–5), or high (≥6).

### Temporal changes of circulating metabolites after MBS

**Figure 1.**
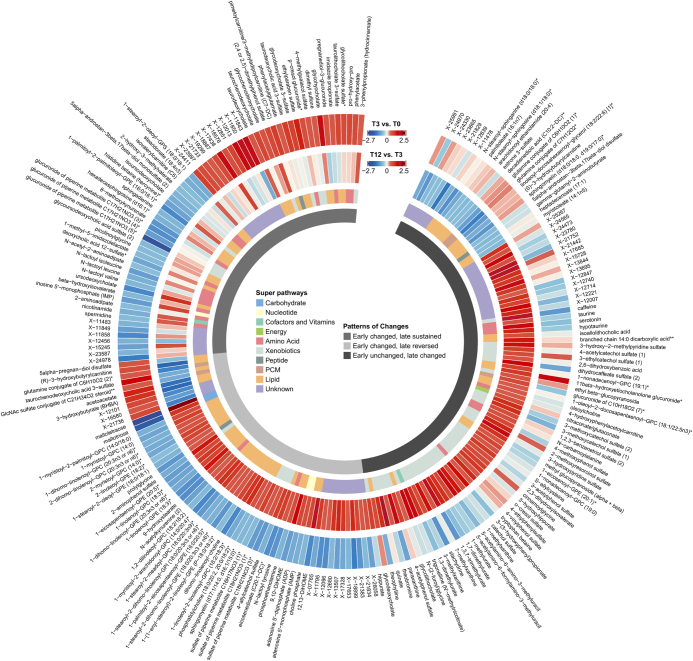
Temporal changes in plasma metabolites after metabolic and bariatric surgery. The circular plot depicts 224 metabolites that exhibited significant and dynamic changes across multiple timepoints. The two outermost rings represent the magnitude of change (log_2_ FC) between consecutive timepoints: T3 vs. T0 (outermost) and T12 vs. T3 (second ring), with color intensity corresponding to the degree of metabolic regulation as indicated in the color scales (red = upregulation, blue = downregulation). The inner two rings categorize each metabolite according to its biochemical classification (super pathway) and temporal patterns of change, respectively (see also Supplemental Digital Content Table 3, available at: http://links.lww.com/JS9/H488 for additional details). Only plasma metabolites with B-H FDR *P* <0.05 and |log_2_ FC| > log_2_ 1.5 were exhibited. T0, pre-surgery; T3, 3-month post-surgery; T12, 12-month post-surgery; FC, fold change; FDR, false discovery rate; PCM, partially characterized molecules.

In GUMMY, we identified 224 circulating metabolites showing significant temporal changes at FDR-*P* < 0.05 and |log_2_ FC| > log_2_ 1.5 (Fig. 1, Supplemental Digital Content Table 3, available at: http://links.lww.com/JS9/H488). Of these, 66 metabolites were early changed and late sustained; 61 early changed but late reversed; and 97 early unchanged but late changed. Metabolites with sustained increases included primary and secondary bile acids, with glycohyocholate demonstrating the most pronounced increase (FC_T3vsT0_ = 3.18, FC_T12vsT3_ = 1.31). Sustainedly decreased metabolites included BCAAs (leucine, isoleucine, and valine), lactoyl amino acids, and lysine metabolites (FC_T3vsT0_ ranges: 0.49–0.67; FC_T12vsT0_ 0.51–0.74). Ketone bodies exhibited early increases but subsequent decreases (e.g., for acetoacetate, FC_T3vsT0_ = 2.47, FC_T12vsT3_ = 0.31). Conversely, phospholipids [phosphatidylcholine (PC) and phosphatidylethanolamine (PE)], certain lysophospholipids, and glycogen metabolites demonstrated early decreases followed by later increases. Among these, glycogen metabolite, maltotetraose, demonstrated the most pronounced temporal dynamics, with the greatest initial decrease followed by the largest late-phase increase (FC_T3vsT0_ = 0.16 and FC_T12vsT3_ = 5.44). Early unchanged and late increased metabolites were predominantly related to taurine, caffeine, benzoate, and certain tryptophan pathways, with caffeine showing the largest late-phase increase (FC_T12vsT3_ = 3.80). Early unchanged and late decreased metabolites included certain ceramides and long-chain monounsaturated fatty acids (LCMUFAs), with ceramide – *N*-stearoyl-sphinganine (d18:0/18:0) – showing the largest late-phase decrease (FC_T12vsT3_ = 0.42).

### Associations between MBS-altered metabolites and incident CHD

**Figure 2.**
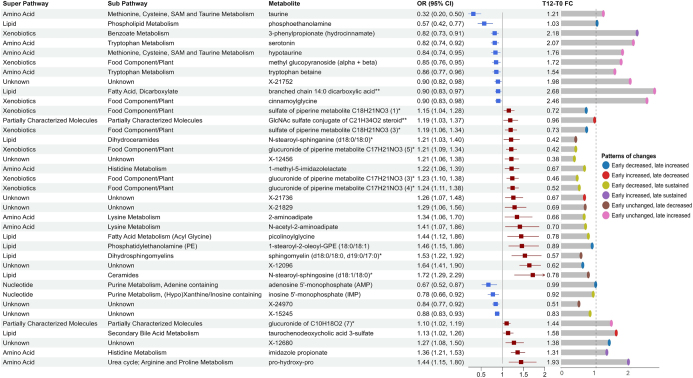
Associations between circulating metabolites significantly changed after metabolic and bariatric surgery and incident CHD risk. The figure shows the bidirectional relationship between 37 metabolites significantly modulated by metabolic and bariatric surgery and incident CHD risk. Left panel: Metabolites categorized by sub-pathways. Right panel: Forest plot depicting ORs with 95% CIs for incident CHD risk per SD increase in each metabolite, with corresponding T12 *vs*. T0 fold changes in GUMMY were shown in lollipop plot. Notably, metabolites with elevated concentrations 1-year post-surgery (FC > 1) were consistently associated with decreased CHD risk (OR < 1.0), while those with reduced post-surgical levels demonstrated positive associations with incident CHD risk. T0, pre-surgery; T12, 12-month post-surgery; FC, fold change; OR, odds ratio; CI, confidence interval; PCM, partially characterized molecules.

Among the 224 circulating metabolites exhibiting substantial changes after MBS, 180 were available in the SCCS samples (Supplemental Digital Content Table 4, available at: http://links.lww.com/JS9/H488). Of these, 37 metabolites were significantly associated with incident CHD [FDR < 0.1 and odds ratio (OR) per SD ≥ 1.1 or ≤ 0.9] and 28 metabolites demonstrated concordant directional effects between post-surgical changes and incident CHD risk (Fig. 2) – metabolites increased at 12-month post-surgery (FC_T12vsT0_ > 1) corresponded with a reduced CHD risk (OR < 1.0), while those decreased post-surgery (FC_T12vsT0_ < 1) were associated with elevated CHD risk (OR > 1.0). The main affected pathways included amino acids (taurine, tryptophan, and lysine metabolism), lipids (ceramides/dihydrosphingomyelin-related metabolites), and xenobiotics. Metabolites involved in taurine and tryptophan pathways, which increased significantly after surgery, demonstrated beneficial associations. For example, a 1-SD increase in taurine was associated with a 68% reduced CHD risk (OR = 0.32 [95% confidence interval (CI) 0.20–0.50]; FC_T12vsT0_ = 1.21). Similar protective effects were observed for hypotaurine, serotonin, and tryptophan betaine. Conversely, metabolites in ceramide and lysine pathways exhibited associations with an increased CHD risk. A 1-SD increase in ceramide – *N*-stearoyl-sphingosine (d18:1/18:0) – was associated with elevated CHD risk (OR = 1.72 [95% CI 1.29–2.29]; FC_T12vsT0_ = 0.78). Similar post-surgery change patterns and CHD risk associations were observed for dihydroceramide *N*-stearoyl-sphinganine (d18:0/18:0), dihydrosphingomyelin (d18:0/18:0, d19:0/17:0), and lysine metabolites 2-aminoadipate and *N*-acetyl-2-aminoadipate.

### Sensitivity analyses

Temporal changes in 224 metabolites remained robust after comprehensive adjustments for covariates (demographics, procedure type, baseline comorbidities, baseline weight, diet quality, and fasting time), with high correlation between unadjusted and adjusted LMM coefficients (*r* = 0.998, *P* < 0.001) (Supplemental Digital Content Figure 2, available at: http://links.lww.com/JS9/H487). Coefficients for metabolite changes per 3-month post-surgery in fully adjusted LMMs are shown in Supplemental Digital Content Table 5, available at: http://links.lww.com/JS9/H488. None of the 224 metabolites showed significant effect modification by sex or race (all FDR-adjusted *p* for interaction > 0.90) (Supplemental Digital Content Table 5, available at: http://links.lww.com/JS9/H488). Most metabolites exhibited consistent FCs by procedure type (RYGB or SG); only two metabolites showed significant interaction effects (FDR < 0.05) between time trajectory and procedure type: myristoleate (14:1n5), an LCMUFA, and X-17438, an unidentified metabolite (Supplemental Digital Content Table 5, available at: http://links.lww.com/JS9/H488). Relaxing the FC threshold to |log_2_FC| > log_2_1.2, we identified an additional 293 metabolites changed after MBS (Supplemental Digital Content Table 6, available at: http://links.lww.com/JS9/H488), including sustained increases in bile acids, cysteine metabolites, serine, and dicarboxylate fatty acids; sustained decreases in certain tryptophan metabolites (kynurenines and kynurenate), phenylalanine metabolites, certain tyrosine metabolites, glutamate, and dihydrosphingomyelins; early increases but late decreases in pregnenolone steroids and sphingomyelins; early decreases but late increases in PC, PE, and certain lysophospholipids; late increases in some lysophospholipids; and late decreases in ceramide metabolites, diacylglycerol lipids, and long-chain polyunsaturated fatty acids (*n*3 and *n*6). In SCCS, similar metabolite–CHD associations persisted after adjusting for BMI, diabetes, hypertension, and dyslipidemia (Supplemental Digital Content Table 7, available at: http://links.lww.com/JS9/H488). No significant interactions were observed between 37 significant metabolites and race, sex, or obesity (all FDR > 0.05). However, potential effect modifications by diabetes, hypertension, and dyslipidemia were observed for tryptophan betaine (all FDR < 0.05). In stratified analyses, tryptophan betaine showed a stronger association among individuals with a history of diabetes, hypertension, or dyslipidemia (all FDR < 0.001) compared to those without corresponding comorbidity (all *P* > 0.1). Xenobiotic methyl glucopyranoside (alpha + beta) and lipid metabolites [sphingomyelin (d18:0/18:0, d19:0/17:0)], *N*-stearoyl-sphingosine (d18:1/18:0), and *N*-stearoyl-sphinganine (d18:0/18:0) showed stronger associations among individuals with dyslipidemia (all FDR < 0.01) compared to those without dyslipidemia (all *P* > 0.1) (Supplemental Digital Content Table 8, available at: http://links.lww.com/JS9/H516).

## Discussion

We performed comprehensive plasma metabolomic profiling in a longitudinal cohort of MBS patients to characterize temporal changes in circulating metabolites. We identified 224 circulating metabolites showing significant temporal changes post-MBS, fallling into three main patterns: early changed, late sustained; early changed, late reversed; and early unchanged, late changed. The altered metabolites primarily included lipids, amino acids, and xenobiotics. Furthermore, we explored associations between surgery-altered metabolites and incident CHD in an independent general population-based prospective nested case–control study and found 28 metabolites significantly associated with incident CHD with concordant effect directions, mainly from lipid pathways (ceramides, PE, fatty acids), amino acid metabolism (lysine and taurine), and xenobiotics (food/plant component metabolism). These findings have important clinical implications, suggesting that MBS-induced alterations in circulating metabolites may contribute to the reduced CHD risk after surgery through specific metabolic pathways. The identified metabolites could potentially serve as novel biomarkers for monitoring cardiovascular risk reduction after MBS and as novel therapeutic targets for CHD prevention in both surgical and non-surgical settings.

### Temporal changes in lipid metabolism after MBS

Consistent with prior research, we observed significant changes in lipids and related metabolites, including sustained increases in some bile acids^[^13,18,38,39^]^, early decreases but late increases in lysophospholipids and phospholipids (PC and PE) at large^[^25,40–42^]^, early increases but late decreases in ketone bodies^[^16,43–45^]^, and late decreases in ceramides and LCMUFAs^[^14,24,46,47^]^. The significant elevation of primary and secondary bile acids after MBS, including glycochenodeoxycholate, taurochenodeoxycholate, glycohyocholate, taurodeoxycholate, and their sulfated derivatives (glycolithocholate sulfate, taurolithocholate 3-sulfate, glycodeoxycholate 3-sulfate, and taurodeoxycholic acid 3-sulfate), reflects alterations in the enterohepatic circulation and gut microbiota metabolism. These bile acids, categorized as primary (hepatically derived) or secondary (microbially modified) based on their biosynthetic origins, exhibited increased plasma concentrations post-surgery, likely due to direct suppression via hepatic farnesoid X receptor-mediated transcriptional inhibition and indirect negative feedback through FGF19-FGFR4 signaling^[^8,13,48^]^. The observed early decrease but late increase in lysophospholipids and phospholipids metabolism, including lysophosphatidylcholines such as 1-myristoyl-GPC (14:0), 1-linolenoyl-GPC (18:3), and lysophosphatidylethanolamines such as 1-eicosapentaenoyl-GPE (20:5), as well as PCs like 1,2-dilinoleoyl-GPC (18:2/18:2), potentially reflect alterations in dietary fat absorption, intestinal microbial metabolism, or hepatic lipid processing induced by MBS^[^41,42^]^. The transient elevation of ketone bodies in the early post-surgery period, followed by a return to near-baseline levels, suggests dynamic metabolic adaptations. Potential mechanisms may involve increased lipolysis and enhanced amino acid catabolism (supported by the concurrent reduction in serum levels of ketogenic amino acids isoleucine, leucine, and phenylalanine). The eventual decline of ketone bodies to approximate pre-surgery levels indicates a transition from acute catabolic response to metabolic stabilization^[^45,49,50^]^. The time-dependent pattern of ceramides/dihydrosphingomyelin metabolites and LCMUFAs (late reduction) likely reflects gradual improvements in inflammatory status and insulin sensitivity^[^25,51^]^.

### Temporal changes in amino acid metabolism after MBS

Sustained reductions in BCAAs, lactoyl amino acids, and lysine metabolites were observed throughout 12 months after MBS. The decline in BCAA levels is consistent with previous studies^[^14,38,40,45,46,50,52,53^]^, which may be attributed to reduced amino acid absorption and enhanced BCAA catabolism^[^16,49^]^. No prior research has reported significant alterations in lactoyl amino acids after MBS, specifically *N*-lactoyl isoleucine, *N*-lactoyl leucine, and *N*-lactoyl valine. However, elevated concentrations of these metabolites have been documented in obese patients with T2D compared to those without^[^54^]^. Lysine metabolites, specifically 2-aminoadipate (i.e., 2-aminoadipic acid, 2-AAA), exhibited sustained post-surgery reductions, with FC_T3vsT0_ of 0.64 and FC_T12vsT3_ of 0.96. These observations align with previous findings from a Norwegian study that showed consistent decreases in 2-AAA concentrations among 41 patients after RYGB between weeks 3 and 9 post-surgery^[^55^]^. Tryptophan-related metabolites (serotonin and tryptophan betaine) demonstrated significant late-phase elevations (FC_T12vsT3_ = 1.54 and 2.07, respectively), which have not been reported in the literature. Sulfur-containing amino acid metabolism exhibited late increases in taurine (FC_T12vsT3_ = 1.67) and hypotaurine (FC_T12vsT3_ = 2.49). However, few studies have reported changes in taurine levels after MBS, with one multicenter study conducted in Norway (*n* = 30) and Sweden (*n* = 30) finding no significant alterations at 6-month and 1-year follow-ups^[^30^]^.

### Temporal changes in xenobiotics after MBS

Additionally, our study revealed distinct temporal patterns in caffeine metabolism after MBS. The slight, non-significant decrease in plasma caffeine metabolites at 3-month post-surgery aligns with previous findings^[^56,57^]^. However, our study uniquely extends the temporal window, revealing a significant upward trend in caffeine metabolites at 12-month post-surgery compared to both baseline and 3-month levels, including paraxanthine, 3-methylxanthine, 5-acetylamino-6-amino-3-methyluracil, and 1-methylxanthine. This pattern, combined with our previous observation of decreased fecal caffeine metabolites and reported findings on enhanced weight loss with caffeine intervention^[^7,58^]^ suggests a progressive adaptation in caffeine metabolism. Potential mechanisms may include enhanced absorption efficiency, reduced resting energy expenditure, and gut microbiota changes^[^7,58^]^. Our findings also revealed that multiple benzoate metabolites showed no significant changes in early post-surgery but marked increases in the late phase. For example, FC_T3vsT0_ and FC_T12vsT3_ for 3-methoxycatechol sulfate (1) were 0.60 and 2.33 in our study, consistent with prior reported significant increases in 3-methoxycatechol sulfate (1) at 1 year after MBS compared to 3 months^[^14^]^. Changed benzoate metabolites are primarily derived from gut microbial metabolism of dietary polyphenols and aromatic amino acids, suggesting that MBS induces progressive alterations in gut microbial composition and activity, particularly in pathways related to aromatic compound metabolism^[^59^]^.

### Associations between MBS-altered metabolites and incident CHD

Among 28 metabolites that demonstrated concordant directional effects between post-surgical changes and incident CHD risk, most were amino acids and lipids. Our research suggested that hypotaurine and taurine, which significantly increased after MBS (respective FC_T12vsT0_ 1.76 and 1.21), were inversely associated with incident CHD, with OR (95% CI) of 0.84 (0.74, 0.95) and 0.32 (0.20, 0.50), respectively. Hypotaurine is a compound derived from cysteine and cysteine sulfinic acid and is oxidized to taurine^[^60–62^]^. Taurine has demonstrated multiple beneficial effects, including anti-inflammatory properties, blood pressure regulation, and enhancement of cardiac function, while also improving glucose homeostasis and lipid metabolism, collectively contributing to improved cardiometabolic health^[^61,63^]^. Our study revealed the beneficial effects of certain tryptophan metabolites on CHD, consistent with previous research. A cohort study (*n* = 1114) found that undetectable plasma serotonin levels (in 17% of participants) were associated with elevated heart and atherosclerotic CVD event risk (adjusted hazard ratios >1.96)^[^64^]^. Similarly, higher serum tryptophan betaine levels correlated with reduced blood pressure and a lower risk of neurodegenerative diseases^[^65,66^]^. Conversely, lysine metabolism and ceramide metabolites demonstrated associations with an increased risk of CHD. Specifically, the lysine metabolite 2-AAA was significantly associated with incident CHD (OR 1.34, 95% CI 1.06–1.70). This observation aligns with prior research^[^67^]^, which identified 2-AAA as a marker of cardiometabolic risk in both healthy individuals and those at high cardiometabolic risk. Ceramides such as *N*-stearoyl-sphingosine (d18:1/18:0) demonstrated a positive association with CHD (OR 1.72, 95% CI 1.29-2.29), consistent with prior research showing that elevated ceramides were associated with incident CVD risk, adverse cardiovascular events, and sudden cardiac death^[^68–70^]^. Reducing ceramide levels may be a potential therapeutic target for the prevention of cardiometabolic disorders.

A few metabolites showed unexpected directional associations between post-surgical changes and CHD risk, including pro-hydroxy-pro (a collagen degradation marker)^[^71^]^ and two gut microbiota-related metabolites: imidazole propionate (a microbial histidine metabolite)^[^72^]^ and taurochenodeoxycholic acid 3-sulfate (a secondary bile acid sulfate conjugate)^[^73^]^. They were elevated post-MBS in the GUMMY and associated with increased CHD risk in the SCCS. Their elevations may reflect catabolic or adaptive processes during rapid weight loss, such as increased collagen turnover^[^74^]^, and changes in gut microbiome composition and metabolism of amino acids and bile acids after gastrointestinal reconstruction^[^75,76^]^. Their associations with incident CHD are consistent with other cohort studies conducted in adults with CVDs (e.g., myocardial infarction, transverse aortic constriction, and heart failure)^[^74,77,78^]^. These findings highlight the complexity of metabolite-disease associations across physiological contexts and warrant future research to verify their changes after MBS and mechanistic studies to understand their contribution to CVD health.

### Strengths, limitations, and future directions

Our study has several notable strengths. First, we conducted a longitudinal follow-up of MBS patients to characterize temporal changes in circulating metabolites over one year post-surgery, applying rigorous criteria for both statistical significance (FDR < 0.05) and effect size (|log_2_FC| > log_2_1.5). Three temporal patterns, distinguishing metabolites with different change patterns, may enhance the biological understanding of short- and long-term metabolic adaptations after MBS. Notably, our study provides novel evidence of distinct temporal alterations in lactoyl amino acids, taurine, and caffeine metabolites. Second, our comprehensive approach utilized untargeted metabolomics profiling of 1199 circulating metabolites, facilitating the discovery of novel biomarkers and biological pathways underlying MBS and cardiometabolic improvements. This extensive metabolomic coverage allowed us to capture diverse biochemical changes across multiple pathways, providing insights into the complex physiological adaptations after MBS and their relationship to cardiovascular outcomes. Third, we examined associations between surgery-altered metabolites and incident CHD in a large, nested case–control study, identifying metabolites that potentially underlie the cardiovascular benefits of MBS and may serve as novel biomarkers for cardiovascular risk assessment.

However, our current study also had several limitations. First, this research was observational, which could introduce bias due to confounding. However, we examined within-person metabolite changes, with each patient’s pre-surgery levels serving as their own control. We also applied sensitivity analyses to compare the coefficients in two LMMs, with and without adjusting for potential confounders, and found a very high correlation coefficient (*r* = 0.998, *P* < 0.001), indicating that metabolite changes were driven by surgery rather than other factors. Second, our stringent statistical approach with FDR correction and FC criteria may have missed potentially important changes; however, we additionally identified 293 moderately altered metabolites. Third, GUMMY participants were predominantly White women, which may limit the generalizability of our findings to more diverse populations. While our interaction analyses did not identify significant effect modification by sex or race, future studies with sufficient sample sizes of non-White patients and male patients are needed. Fourth, 44 metabolites that changed after MBS were not detected in the SCCS samples, which need to be evaluated in future studies. Fifth, despite comparable BMI values (SCCS: 30.32 kg/m^2^, GUMMY 12-month post-surgery: 32.62 kg/m^2^), these cohorts reached metabolic states differently. GUMMY patients experienced rapid, surgery-driven metabolic changes (particularly within 3 months), while SCCS participants likely had stable metabolic profiles at the time of cohort enrollment [mean (SD) years before CHD diagnosis: 5.7 (3.7)]. The complementary nature of GUMMY (interventional, short-term, mechanistic) and SCCS (observational, long-term, outcome-focused) enabled us to elucidate both the temporal dynamics of metabolite changes after MBS and their potential implications for long-term CVD outcomes. Associations between MBS-altered metabolites and incident CHD in an independent general population-based cohort strengthen their biological plausibility and generalizability beyond bariatric surgery patients. Nevertheless, the differences between the surgical and non-surgical cohorts support future research to evaluate whether these metabolites are predictive of incident CHD for post-MBS patients. Before sufficient incident CVD events accumulate in surgical patient cohorts, estimated CVD risks using PREVENT equations^[^79^]^ may help assess whether surgery-induced metabolite changes can serve as biomarkers for post-MBS cardiovascular benefit prediction. Lastly, experimental validation of key metabolite-disease associations is essential to establish causality and identify potential therapeutic targets for metabolic intervention beyond surgery.

## Conclusions

In conclusion, our study highlights the temporal changes in circulating metabolites after MBS and identifies surgery-altered metabolites significantly associated with incident CHD. Our findings could improve the understanding of metabolomic changes and cardiometabolic benefits after MBS, which may be applicable to CVD prevention.

## Data Availability

The data used in the GUMMY and SCCS are available from the corresponding author upon reasonable request and committee approval.

## Research registration unique identifying number (UIN)

NCT05000996 ClinicalTrials.gov

## References

[1] GBD. Global, regional, and national prevalence of adult overweight and obesity, 1990-2021, with forecasts to 2050: a forecasting study for the global burden of disease study 2021. Lancet 2025;405:813–38. 40049186 10.1016/S0140-6736(25)00355-1 PMC11920007

[2] Emmerich SD Fryar CD Stierman B. Obesity and severe obesity prevalence in adults: United States, August 2021–August 2023. NCHS Data Brief 2024;508. doi:10.15620/cdc/159281. PMC11744423 39808758

[3] Flegal KM Carroll MD Ogden CL. Prevalence and trends in obesity among US adults, 1999-2000. JAMA 2002;288:1723–27. 12365955 10.1001/jama.288.14.1723

[4] Powell-Wiley TM Poirier P Burke LE. Obesity and cardiovascular disease: a scientific statement from the American Heart Association. Circulation 2021;143:e984–e1010. 33882682 10.1161/CIR.0000000000000973 PMC8493650

[5] Rubino F Cummings DE Eckel RH. Definition and diagnostic criteria of clinical obesity. Lancet Diabetes Endocrinol 2025;13:221–62. 39824205 10.1016/S2213-8587(24)00316-4 PMC11870235

[6] Wang L O’Brien MT Zhang X. Cardiometabolic improvements after metabolic surgery and related presurgery factors. J Endocr Soc 2024;8:bvae027. 38487212 10.1210/jendso/bvae027 PMC10939051

[7] Yu D Shu XO Howard EF. Fecal metagenomics and metabolomics reveal gut microbial changes after bariatric surgery. Surg Obes Relat Dis 2020;16:1772–82. 32747219 10.1016/j.soard.2020.06.032 PMC9057387

[8] Albaugh VL Banan B Antoun J. Role of Bile Acids and GLP-1 in mediating the metabolic improvements of bariatric surgery. Gastroenterology 2019;156:1041–51.e4. 30445014 10.1053/j.gastro.2018.11.017 PMC6409186

[9] Perdomo CM Cohen RV Sumithran P. Contemporary medical, device, and surgical therapies for obesity in adults. Lancet 2023;401:1116–30. 36774932 10.1016/S0140-6736(22)02403-5

[10] Wang L Zhang X Chen Y. Reduced risk of cardiovascular diseases after bariatric surgery based on the new predicting risk of cardiovascular disease EVENTs equations. J Am Heart Assoc 2025;14:e038191. 40055867 10.1161/JAHA.124.038191 PMC12132719

[11] van Veldhuisen SL Gorter TM van Woerden G. Bariatric surgery and cardiovascular disease: a systematic review and meta-analysis. Eur Heart J 2022;43:1955–69. 35243488 10.1093/eurheartj/ehac071 PMC9123239

[12] Akalestou E Miras AD Rutter GA. Mechanisms of weight loss after obesity surgery. Endocr Rev 2022;43:19–34. 34363458 10.1210/endrev/bnab022 PMC8755990

[13] Albaugh VL Banan B Ajouz H. Bile acids and bariatric surgery. Mol Aspects Med 2017;56:75–89. 28390813 10.1016/j.mam.2017.04.001 PMC5603298

[14] Bagheri M Tanriverdi K Iafrati MD. Characterization of the plasma metabolome and lipidome in response to sleeve gastrectomy and gastric bypass surgeries reveals molecular patterns of surgical weight loss. Metabolism 2024;158:155955. 38906372 10.1016/j.metabol.2024.155955 PMC11755375

[15] Bastings J Venema K Blaak EE. Influence of the gut microbiota on satiety signaling. Trends Endocrinol Metab 2023;34:243–55. 36870872 10.1016/j.tem.2023.02.003

[16] Vaz M Pereira SS Monteiro MP. Metabolomic signatures after bariatric surgery - a systematic review. Rev Endocr Metab Disord 2022;23:503–19. 34855133 10.1007/s11154-021-09695-5 PMC9156502

[17] Samczuk P Hady HR Adamska-Patruno E. In-and-out molecular changes linked to the type 2 diabetes remission after bariatric surgery: an influence of gut microbes on mitochondria metabolism. Int J Mol Sci 2018;19:3744. 30477251 10.3390/ijms19123744 PMC6321270

[18] Albaugh VL Flynn CR Cai S. Early increases in bile acids post roux-en-Y gastric bypass are driven by insulin-sensitizing, secondary bile acids. J Clin Endocrinol Metab 2015;100:E1225–33. 26196952 10.1210/jc.2015-2467 PMC4570157

[19] Ikeda T Aida M Yoshida Y. Alteration in faecal bile acids, gut microbial composition and diversity after laparoscopic sleeve gastrectomy. Br J Surg 2020;107:1673–85. 32432347 10.1002/bjs.11654

[20] Ma Q Li Y Li P. Research progress in the relationship between type 2 diabetes mellitus and intestinal flora. Biomed Pharmacother 2019;117:109138. 31247468 10.1016/j.biopha.2019.109138

[21] Wang R Mijiti S Xu Q. The potential mechanism of remission in type 2 diabetes mellitus after vertical sleeve gastrectomy. Obes Surg 2024;34:3071–83. 38951388 10.1007/s11695-024-07378-z

[22] Ilhan ZE DiBaise JK Dautel SE. Temporospatial shifts in the human gut microbiome and metabolome after gastric bypass surgery. NPJ Biofilms Microbiomes 2020;6:12. 32170068 10.1038/s41522-020-0122-5 PMC7070067

[23] Shi Q Wang Q Zhong H. Roux-en-Y gastric bypass improved insulin resistance via alteration of the human gut microbiome and alleviation of endotoxemia. Biomed Res Int 2021;2021:5554991. 34337024 10.1155/2021/5554991 PMC8294027

[24] Huang H Kasumov T Gatmaitan P. Gastric bypass surgery reduces plasma ceramide subspecies and improves insulin sensitivity in severely obese patients. Obesity (Silver Spring) 2011;19:2235–40. 21546935 10.1038/oby.2011.107 PMC3809956

[25] Kayser BD Lhomme M Dao MC. Serum lipidomics reveals early differential effects of gastric bypass compared with banding on phospholipids and sphingolipids independent of differences in weight loss. Int J Obes Lond 2017;41:917–25. 28280270 10.1038/ijo.2017.63

[26] Thaker VV Kwee LC Chen H. Metabolite signature of diabetes remission in individuals with obesity undergoing weight loss interventions. Obesity (Silver Spring) 2024;32:304–14. 37962326 10.1002/oby.23943 PMC11201087

[27] Zhao S Hörkkö S Savolainen MJ. Short-term metabolic changes and their physiological mediators in the roux-en-Y gastric bypass bariatric surgery. Obes Surg 2024;34:625–34. 38191968 10.1007/s11695-023-07042-y PMC10810963

[28] Cuomo P Capparelli R Iannelli A. Role of Branched-chain amino acid metabolism in type 2 diabetes, obesity, cardiovascular disease and non-alcoholic fatty liver disease. Int J Mol Sci 2022;23:4325. 35457142 10.3390/ijms23084325 PMC9030262

[29] Pan XF Chen ZZ Wang TJ. Plasma metabolomic signatures of obesity and risk of type 2 diabetes. Obesity (Silver Spring) 2022;30:2294–306. 36161775 10.1002/oby.23549 PMC9633360

[30] Aasheim ET Elshorbagy AK Diep LM. Effect of bariatric surgery on sulphur amino acids and glutamate. Br J Nutr 2011;106:432–40. 21554803 10.1017/S0007114511000201

[31] Agha RA Ginimol M Rasha R. Revised Strengthening the Reporting of Cohort, Cross-Sectional and Case-Control Studies in Surgery (STROCSS) guideline: an update for the age of Artificial Intelligence. Prem J Sci 2025;10:100081.

[32] Signorello LB Hargreaves MK Steinwandel MD. Southern community cohort study: establishing a cohort to investigate health disparities. J Natl Med Assoc 2005;97:972–79. 16080667 PMC2569308

[33] Deng K Gupta DK Shu XO. Metabolite signature of life’s essential 8 and risk of coronary heart disease among low-income black and white Americans. Circ Genom Precis Med 2023;16:e004230. 38014580 10.1161/CIRCGEN.123.004230 PMC10843634

[34] Ford L Kennedy AD Goodman KD. Precision of a clinical metabolomics profiling platform for use in the identification of inborn errors of metabolism. J Appl Lab Med 2020;5:342–56. 32445384 10.1093/jalm/jfz026

[35] Evans AM DeHaven CD Barrett T. Integrated, nontargeted ultrahigh performance liquid chromatography/electrospray ionization tandem mass spectrometry platform for the identification and relative quantification of the small-molecule complement of biological systems. Anal Chem 2009;81:6656–67. 19624122 10.1021/ac901536h

[36] Xia J Psychogios N Young N. MetaboAnalyst: a web server for metabolomic data analysis and interpretation. Nucleic Acids Res 2009;37:W652–60. 19429898 10.1093/nar/gkp356 PMC2703878

[37] Xia J Sinelnikov IV Han B. MetaboAnalyst 3.0–making metabolomics more meaningful. Nucleic Acids Res 2015;43:W251–7. 25897128 10.1093/nar/gkv380 PMC4489235

[38] Fiamoncini J Fernandes Barbosa C Arnoni Junior JR. Roux-en-Y gastric bypass surgery induces distinct but frequently transient effects on acylcarnitine, bile acid and phospholipid levels. Metabolites 2018;8:83. 30477108 10.3390/metabo8040083 PMC6316856

[39] Yu H Ni Y Bao Y. Chenodeoxycholic acid as a potential prognostic marker for roux-en-Y gastric bypass in Chinese obese patients. J Clin Endocrinol Metab 2015;100:4222–30. 26425885 10.1210/jc.2015-2884

[40] Henning T Kochlik B Kusch P. Pre-operative assessment of micronutrients, amino acids, phospholipids and oxidative stress in bariatric surgery candidates. Antioxidants (Basel) 2022;11:774. 35453460 10.3390/antiox11040774 PMC9031169

[41] Carlsson ER Allin KH Madsbad S. Phosphatidylcholine and its relation to apolipoproteins A-1 and B changes after Roux-en-Y gastric bypass: a cohort study. Lipids Health Dis 2019;18:169. 31488158 10.1186/s12944-019-1111-7 PMC6729082

[42] Lopes TI Geloneze B Pareja JC. Blood metabolome changes before and after bariatric surgery: a (1)H NMR-based clinical investigation. Omics 2015;19:318–27. 25871626 10.1089/omi.2015.0009

[43] Umemura A Sasaki A Kumagai H. Relationships between changes in serum ketone body levels and metabolic effects in patients with severe obesity who underwent laparoscopic sleeve gastrectomy. Obes Surg 2024;34:2607–16. 38842760 10.1007/s11695-024-07337-8 PMC11217106

[44] Angelidi AM Kokkinos A Sanoudou D. Early metabolomic, lipid and lipoprotein changes in response to medical and surgical therapeutic approaches to obesity. Metabolism 2023;138:155346. 36375643 10.1016/j.metabol.2022.155346

[45] Gralka E Luchinat C Tenori L. Metabolomic fingerprint of severe obesity is dynamically affected by bariatric surgery in a procedure-dependent manner. Am J Clin Nutr 2015;102:1313–22. 26581381 10.3945/ajcn.115.110536

[46] Mutch DM Fuhrmann JC Rein D. Metabolite profiling identifies candidate markers reflecting the clinical adaptations associated with Roux-en-Y gastric bypass surgery. PLoS One 2009;4:e7905. 19936240 10.1371/journal.pone.0007905 PMC2775672

[47] Mikhalkova D Holman SR Jiang H. Bariatric surgery-induced cardiac and lipidomic changes in obesity-related heart failure with preserved ejection fraction. Obesity (Silver Spring) 2018;26:284–90. 29243396 10.1002/oby.22038 PMC5783730

[48] Li S Hsu DD Li B. Cytoplasmic tyrosine phosphatase Shp2 coordinates hepatic regulation of bile acid and FGF15/19 signaling to repress bile acid synthesis. Cell Metab 2014;20:320–32. 24981838 10.1016/j.cmet.2014.05.020 PMC4365973

[49] Tulipani S Griffin J Palau-Rodriguez M. Metabolomics-guided insights on bariatric surgery versus behavioral interventions for weight loss. Obesity (Silver Spring) 2016;24:2451–66. 27891833 10.1002/oby.21686

[50] Laferrère B Reilly D Arias S. Differential metabolic impact of gastric bypass surgery versus dietary intervention in obese diabetic subjects despite identical weight loss. Sci Transl Med 2011;3:80re2. 10.1126/scitranslmed.3002043 PMC3656497 21525399

[51] Luukkonen PK Zhou Y Sädevirta S. Hepatic ceramides dissociate steatosis and insulin resistance in patients with non-alcoholic fatty liver disease. J Hepatol 2016;64:1167–75. 26780287 10.1016/j.jhep.2016.01.002

[52] Lips MA Van Klinken JB van Harmelen V. Roux-en-Y gastric bypass surgery, but not calorie restriction, reduces plasma branched-chain amino acids in obese women independent of weight loss or the presence of type 2 diabetes. Diabetes Care 2014;37:3150–56. 25315204 10.2337/dc14-0195

[53] Modesitt SC Hallowell PT Slack-Davis JK. Women at extreme risk for obesity-related carcinogenesis: baseline endometrial pathology and impact of bariatric surgery on weight, metabolic profiles and quality of life. Gynecol Oncol 2015;138:238–45. 26013696 10.1016/j.ygyno.2015.05.015

[54] Scott B Day EA O’Brien KL. Metformin and feeding increase levels of the appetite-suppressing metabolite Lac-Phe in humans. Nat Metab 2024;6:651–58. 38499765 10.1038/s42255-024-01018-7 PMC11052712

[55] Karlsson C Johnson LK Greasley PJ. Gastric bypass vs diet and cardiovascular risk factors: a nonrandomized controlled trial. JAMA Surg 2024;159:971–80. 38959017 10.1001/jamasurg.2024.2162 PMC11223056

[56] Goday Arno A Farré M Rodríguez-Morató J. Pharmacokinetics in morbid obesity: influence of two bariatric surgery techniques on paracetamol and caffeine metabolism. Obes Surg 2017;27:3194–201. 28560524 10.1007/s11695-017-2745-z

[57] Li S Shi C Wu H. Longitudinal changes of serum metabolomic profile after laparoscopic sleeve gastrectomy in obesity. Endocr Connect 2024;13:e240292. 39302038 10.1530/EC-24-0292 PMC11562687

[58] Rebello CJ Greenway FL Zhang D. Sympathomimetic increases resting energy expenditure following bariatric surgery: a randomized controlled clinical trial. Obesity (Silver Spring) 2022;30:874–83. 35244344 10.1002/oby.23384 PMC10167942

[59] Hertel J Fässler D Heinken A. NMR metabolomics reveal urine markers of microbiome diversity and identify benzoate metabolism as a mediator between high microbial alpha diversity and metabolic health. Metabolites 2022;12:308. 35448495 10.3390/metabo12040308 PMC9025190

[60] Wójcik OP Koenig KL Zeleniuch-Jacquotte A. The potential protective effects of taurine on coronary heart disease. Atherosclerosis 2010;208:19–25. 19592001 10.1016/j.atherosclerosis.2009.06.002 PMC2813349

[61] Qaradakhi T Gadanec LK McSweeney KR. The anti-inflammatory effect of taurine on cardiovascular disease. Nutrients 2020;12:2847. 32957558 10.3390/nu12092847 PMC7551180

[62] Tzang CC Lin WC Lin LH. Insights into the cardiovascular benefits of taurine: a systematic review and meta-analysis. Nutr J 2024;23:93. 39148075 10.1186/s12937-024-00995-5 PMC11325608

[63] Münzker J Haase N Till A. Functional changes of the gastric bypass microbiota reactivate thermogenic adipose tissue and systemic glucose control via intestinal FXR-TGR5 crosstalk in diet-induced obesity. Microbiome 2022;10:96. 35739571 10.1186/s40168-022-01264-5 PMC9229785

[64] Edmonston D Isakova T Wolf M. Plasma serotonin and cardiovascular outcomes in chronic kidney disease. J Am Heart Assoc 2023;12:e029785. 37609990 10.1161/JAHA.123.029785 PMC10547345

[65] Kim H Appel LJ Lichtenstein AH. Metabolomic profiles associated with blood pressure reduction in response to the DASH and DASH-sodium dietary interventions. Hypertension 2023;80:1494–506. 37161796 10.1161/HYPERTENSIONAHA.123.20901 PMC10262995

[66] Zhang A Pan C Wu M. Causal association between plasma metabolites and neurodegenerative diseases. Prog Neuropsychopharmacol Biol Psychiatr 2024;134:111067. 10.1016/j.pnpbp.2024.111067 38908505

[67] Desine S Gabriel CL Smith HM. Association of alpha-aminoadipic acid with cardiometabolic risk factors in healthy and high-risk individuals. Front Endocrinol (Lausanne) 2023;14:1122391. 37745703 10.3389/fendo.2023.1122391 PMC10513411

[68] Meeusen JW Donato LJ Bryant SC. Plasma Ceramides. Arteriosclerosis Thrombosis Vasc Biol 2018;38:1933–39. 10.1161/ATVBAHA.118.311199 29903731

[69] Wang DD Toledo E Hruby A. Plasma Ceramides, Mediterranean Diet, and Incident Cardiovascular Disease in the PREDIMED Trial (Prevención con Dieta Mediterránea). Circulation 2017;135:2028–40. 28280233 10.1161/CIRCULATIONAHA.116.024261 PMC5496817

[70] Bockus LB Jensen PN Fretts AM. Plasma ceramides and sphingomyelins and sudden cardiac death in the cardiovascular health study. JAMA Network Open 2023;6:e2343854. 37976059 10.1001/jamanetworkopen.2023.43854 PMC10656644

[71] Garcia CK Gambino BJ Robinson GP. Delayed metabolic disturbances in the myocardium after exertional heat stroke: contrasting effects of exertion and thermal load. J Appl Physiol (1985) 2023;135:1186–98. 37795530 10.1152/japplphysiol.00372.2023 PMC10979828

[72] Zhu L Li J Li Y. Histidine intake, human gut microbiome, plasma levels of imidazole propionate, and coronary heart disease risk in US Adults. Curr Dev Nutr 2022;6:1041.

[73] Mao F Liu T Hou X. Increased sulfation of bile acids in mice and human subjects with sodium taurocholate cotransporting polypeptide deficiency. J Biol Chem 2019;294:11853–62. 31201272 10.1074/jbc.RA118.007179 PMC6682732

[74] Sansbury BE DeMartino AM Xie Z. Metabolomic analysis of pressure-overloaded and infarcted mouse hearts. Circulation 2014;7:634–42. 10.1161/CIRCHEARTFAILURE.114.001151 PMC4102656 24762972

[75] Chen Y Chaudhari SN Harris DA. A small intestinal bile acid modulates the gut microbiome to improve host metabolic phenotypes following bariatric surgery. Cell Host Microbe 2024;32:1315–30.e5. 39043190 10.1016/j.chom.2024.06.014 PMC11332993

[76] Gopalakrishnan V Kumar C Robertsen I. A multi-omics microbiome signature is associated with the benefits of gastric bypass surgery and is differentiated from diet induced weight loss through 2 years of follow-up. Mucosal Immunol 2025;18:825–35. 40222615 10.1016/j.mucimm.2025.04.002

[77] Molinaro A Nemet I Bel Lassen P. Microbially produced imidazole propionate is associated with heart failure and mortality. JACC Heart Fail 2023;11:810–21. 37115134 10.1016/j.jchf.2023.03.008 PMC12512386

[78] Lejonberg C Czuba T Egerstedt A. Metabolite Profiles of heart failure, central hemodynamic derangement, and response to heart transplantation. J Am Heart Assoc 2025;14:e039248. 40654253 10.1161/JAHA.124.039248 PMC12533607

[79] Khan SS Matsushita K Sang Y. Development and validation of the American Heart Association’s PREVENT equations. Circulation 2024;149:430–49. 37947085 10.1161/CIRCULATIONAHA.123.067626 PMC10910659

